# Association of Skeletal Muscle Strength with Cognitive Performance After Metabolic Bariatric Surgery

**DOI:** 10.3390/jcm15020818

**Published:** 2026-01-20

**Authors:** María-José Barahona, Andreu Simó-Servat, Montse Ibarra, Mireia Libran, Verónica Perea, Judith Castejón, Carlos Puig-Jové, Carmen Quirós, Laura Casas

**Affiliations:** 1 Department of Endocrinology and Nutrition, Hospital Universitari Mútua Terrassa, Plaça del Doctor Robert 5, 08221 Terrassa, Spain; asimo@mutuaterrassa.cat (A.S.-S.); mibarra@mutuaterrassa.cat (M.I.); mlibran@mutuaterrassa.es (M.L.); vperea@mutuaterrassa.cat (V.P.); cpuig@mutuaterrassa.cat (C.P.-J.); cquiros@mutuaterrassa.cat (C.Q.); 2 Department of Neurology, Hospital Universitari Mútua Terrassa, Plaça del Doctor Robert, 5, 08221 Terrassa, Spain; jcastejon@mutuaterrassa.cat (J.C.); lcasas@mutuaterrassa.cat (L.C.)

**Keywords:** metabolic bariatric surgery, cognitive function, handgrip strength, appendicular lean mass, neurocognitive test battery

## Abstract

**Background/Objectives:** Obesity is associated with cognitive decline, and metabolic bariatric surgery (MBS) can improve both physical and cognitive outcomes. However, cognitive improvements post-surgery are variable. This study explores the role of skeletal muscle health, specifically muscle strength and mass, in cognitive performance after MBS, aiming to identify factors that influence cognitive recovery. **Methods:** In this prospective study, 55 patients scheduled for MBS were assessed 1 month before and 12 months after surgery. Body composition, including appendicular lean mass (ALM) and fat mass, was measured using dual-energy X-ray absorptiometry (DXA). Handgrip strength (HGS) was assessed via dynamometry, and neurocognitive performance was evaluated using a standardized test battery. **Results:** Significant improvements in cognitive performance were observed at 12 months post-surgery in attention, memory, language, executive functions and overall cognitive performance. Regarding body composition, significant reductions were observed in fat mass and body mass index (BMI). A decrease in ALM and no changes in HGS were observed; however, when adjusted for body size, both showed an increase. Regression analysis identified baseline HGS, but not muscle mass, as a significant predictor of cognitive performance at 12 months post-surgery. **Conclusions:** MBS leads to significant improvements in cognitive function. Our results suggest that baseline HGS may play a role in predicting cognitive outcomes post-surgery, highlighting the need for comprehensive assessments of both physical and cognitive health in patients undergoing MBS. Further longitudinal studies are needed to explore causal relationships and the potential reversibility of cognitive deficits post-surgery.

## 1. Introduction

Obesity is a global health issue defined by the World Health Organization (WHO) as an excessive accumulation of fat that increases the risk of several comorbidities, including diabetes, cardiovascular diseases, and certain cancers [1,2,3,4]. It is also recognized as a major risk factor for cognitive decline, with studies linking obesity to neurological disorders such as Alzheimer’s disease and vascular dementia [5,6,7,8,9,10,11]. Obesity has been associated with decreased cognitive performance, mainly in tasks involving attention, information processing speed, memory and executive functions such as tasks that require inhibition, cognitive flexibility, working memory, decision making, verbal fluency and planning when compared to individuals of normal weight [12,13,14,15,16]. Over the years, researchers have been striving to identify the biological mechanisms underlying the connection between obesity and cognitive impairment. Although the exact nature of this association is yet to be determined, numerous pathways have been suggested which encompass inflammation, vascular damage, metabolic mechanisms, hormonal imbalances and alterations in the gut microbiome, among others [17,18,19]. Additionally, obesity-related conditions such as sleep apnea and diabetes have cognitive consequences [20,21].

Metabolic bariatric surgery (MBS) is an effective intervention for severe obesity (SO), leading to significant improvements in physical health outcomes such as weight loss and metabolic parameters. Emerging evidence shows that postoperative weight loss is associated with improvements in cognitive performance [22,23,24,25,26,27]. For instance, a recent meta-analysis found significant post-surgical gains in attention, memory and executive function, though language abilities showed no significant change [28]. Likewise, a longitudinal study observed marked improvements in cognitive scores (particularly in attention and verbal fluency) within 6 months after surgery, which persisted at 24 months [29]. However, not all cognitive domains improve uniformly; some reports highlight stabilization (no further decline) of overall cognitive function rather than dramatic enhancement in the years following MBS [30]. These mixed outcomes underscore the need to identify factors that influence cognitive trajectories in post-bariatric patients.

One such factor may be skeletal muscle health. The loss of muscle mass and function, also known as sarcopenia, has garnered attention for its potential role in cognitive decline. Numerous studies indicate that sarcopenia is associated with poorer cognitive function and a higher prevalence of cognitive impairment in older adults [31]. Notably, recent findings suggest that the functional component of sarcopenia (e.g., low muscle strength or poor physical performance) is more strongly linked to cognitive deficits than low muscle mass alone [32,33]. In other words, muscle function seems to drive the association between sarcopenia and cognitive health, whereas muscle mass per se plays a smaller role. This could be particularly relevant for patients with obesity undergoing MBS: many patients are middle-aged or older and may already have pre-existing muscle weakness or low muscle quality despite high body weight. Rapid weight loss after surgery can further alter body composition, raising the risk of or unmasking sarcopenia [34,35].

Given the connections between muscle health and cognition, the role of skeletal muscle strength and mass in cognitive outcomes after MBS warrants special attention. Improvements in neurocognitive function post-surgery might depend not only on weight loss and metabolic factors but also on the preservation or enhancement of muscle function. In this context, the present study examines the association of skeletal muscle parameters (muscle strength and mass) with cognitive performance after MBS.

## 2. Materials and Methods

This was a prospective study conducted at our hospital. Patients eligible for MBS were those with a body mass index (BMI) > 35 kg/m^2^ and comorbidities or BMI > 40 kg/m^2^ selected from the SO Unit. A total of 55 patients undergoing Y-de-Roux gastric bypass were recruited from those attending the MBS program at our hospital between January 2020 and November 2021. Body composition was assessed using dual-energy X-ray absorptiometry (DXA; General Electric, GE Healthcare, Chicago, IL, USA), handgrip strength (HGS) was measured using a dynamometer (Jamar Plus Digital Hand Dynamometer, Performance Health, Nottinghamshire, UK) and neurocognitive function was evaluated with the neurocognitive test battery (NTB) administered to all patients 1 month prior and 12 months after MBS. Additionally, baseline and post-surgery blood tests were performed to establish the glycemic and lipid profile. Quality of life (QoL) was evaluated using the Moorehead–Ardelt questionnaire.

Patients did not participate in any structured exercise or resistance training program during the study period. Regarding dietary recommendations, prior to MBS, patients were advised to follow a normoproteic Mediterranean diet (0.8–1.2 g/kg/day of protein). After MBS, once oral intake was adequately tolerated, patients were advised to increase protein intake to 1.2–1.5 g/kg/day.

Exclusion criteria included individuals over 65 years, pregnant women, patients with clinical or personal characteristics that make monitoring difficult (e.g., drug or alcohol addiction, severe psychological or psychiatric disorders) and patients with a history of trauma injury, spine injury or any process that could impair motor function in the limbs.

The study was approved by the local Ethics Committee of the Fundació Assistencial Mútua Terrassa Comité Ético de Investigación con Medicamentos (protocol code Acta 08/2019 and 2 October 2019). It was conducted in accordance with the ethical standards of the institutional research committee and with the Declaration of Helsinki “Ethical Principles for Medical Research Involving Human Subjects”. Informed consent was obtained from all participants included in the study.

### 2.1. Neuropsychological Assessment

A well-validated, comprehensive standardized neurocognitive battery of tests lasting approximately 1 h was administered to all participants. The tests assessed different cognitive domains including attention, memory (both verbal and visual), psychomotor performance and speed, language, executive function and visuospatial praxis. Verbal attention was evaluated using the forward digit span (a list of numbers that a person must repeat in the correct order immediately after presentation), and visual attention was assessed using the Corsi block-tapping test (it involves mimicking a researcher as they tap a sequence of up to nine identical spatially separated blocks). Psychomotor speed was measured using the total score of the Symbol Digit Modalities Test (SDMT), which requires filling in the boxes with the appropriate number according to the symbols, and the Trail Making Test (TMT)-A, where participants must connect a set of numbers in the correct sequence. Language abilities were assessed using the Boston Naming Test (BNT) for denomination in which subjects were asked to name pictures. Verbal learning and memory were assessed using the Free and Cued Selective Reminding Test (FCSRT), which involves recalling words with or without semantic cues, while visual construction and memory were measured using the Rey Complex Figure Test (RCFT), which requires reproducing and recalling a complex figure. We used Grooved Pegboard for manual dexterity and bimanual coordination. Executive functions were evaluated using the TMT-B (which requires participants to connect a set of letters and numbers in the correct order), the Stroop Color and Word Test (which measures the ability to inhibit automatic cognitive processes where participants must name the ink color rather than the printed color), and the Block Design Test (which involves the participant arranging colored blocks to match a given pattern or design). In addition, verbal and visual working memory were assessed using the backward digit span (a list of numbers that a person must recall in reverse order) and the Backward Corsi Task (which involves presenting a sequence of blocks that the participant must tap in reverse order after observing the researcher’s actions), respectively. Finally, verbal fluency was assessed through two tasks: Category Verbal Fluency (where subjects must name as many animals as possible) and Phonological Verbal Fluency (where subjects must name as many words as possible beginning with the letter “P”).

Raw scores for each cognitive test were initially obtained and then transformed into scale scores adjusted for age and education level. These scale scores were subsequently converted to z-scores using the mean and standard deviation of the relevant normative population. To create domain-specific composite scores, the z-scores for each test within a domain were averaged. These domain composite scores were then combined to generate a global cognitive composite score. An experienced neuropsychologist (L.C.) and a well-trained research psychologist (J.C.) administered the neuropsychological tests, which were all conducted in a quiet environment at the hospital.

### 2.2. Body Composition by DXA

The variables that were obtained related to body composition were as follows: appendicular lean mass (ALM), ALM adjusted to body weight (ALM/W), and trunk and total fat mass measured by DXA using a General Electric (GE Healthcare) Prodigy Advance model, software EnCore version 12.30.

### 2.3. Dynamometer

HGS was assessed using a Jamar Plus Digital Hand Dynamometer. The patient was seated in an anatomical position on a chair with back support and fixed armrests, ensuring the forearm was flexed at 90° without contact with the torso. Patients were instructed to grip the dynamometer with maximum effort and maintain the grip for as long as possible, alternating between the right and left hands. Three readings were taken for each hand, with a 10–20 s rest between attempts to minimize muscle fatigue. The highest value in kilograms for each hand was recorded as the result.

### 2.4. Statistical Analysis

Continuous variables are expressed as the mean ± standard deviation (SD) if normally distributed or as the median with an interquartile range (IQR) if non-normally distributed. Categorical variables are expressed as percentages. Differences between groups were assessed using the chi-squared test for categorical variables, Student’s *t*-test for continuous variables with normal distribution and the Mann–Whitney U test for non-normally distributed continuous variables. To identify predictors of cognitive performance, multiple regression analyses were performed. Statistical analyses were conducted using IBM SPSS Statistics version 25.0. A *p*-value < 0.05 was considered statistically significant.

## 3. Results

The following tables provide key information on the participants’ characteristics and clinical data. Table 1 presents the baseline characteristics of the participants, and Table 2 displays the laboratory parameters related to the lipid and glycemic profiles at baseline and 12 months post-surgery. Significant improvements were observed in total cholesterol, HDL-cholesterol, LDL-cholesterol, triglycerides, plasma glucose, insulin levels, homeostatic model assessment for insulin resistance (HOMA-IR), and HbA1c, with all values showing statistically significant changes (*p* < 0.001). These results indicate a marked improvement in lipid and glycemic parameters following MBS.

Table 3 summarizes the changes in body composition and quality of life (QoL) from baseline to 12 months after surgery. Significant improvements were observed in body composition, including a marked reduction in BMI, trunk and total fat mass. ALM also decreased; however, when adjusted for body weight, it increased. Muscle strength measured by the dynamometer did not change, but it increased when adjusted for BMI. Regarding quality of life, an improvement was observed at 12 months post-surgery.

Table 4 presents the neurocognitive results at baseline and 12 months post-surgery across six cognitive domains, with the corresponding neurocognitive tests belonging to each domain listed in the left column. Significant improvements were observed in the domains of attention/processing speed, memory, language and executive function. On the other hand, no significant changes were observed in the psychomotor speed domain, as assessed by the Grooved Pegboard Test, and in the visuospatial praxis domain, evaluated using the ROCF Copy test. The global composite score, which represents the overall cognitive performance, also showed a significant improvement (*p* < 0.001). These results suggest that MBS leads to significant cognitive improvements in attention, memory, language and executive function, while psychomotor speed and visuospatial praxis remain unaffected after 12 months.

A multivariable linear regression analysis, including variables such as sex, age, educational level, baseline HGS, baseline ALM and HBa1c levels, was performed. In the attention domain at 12 months after MBS, baseline HGS showed a significant independent association (B = 0.051, 95% CI 0.013 to 0.088, *p* = 0.009). Age was also associated with attention performance (B = 0.044, 95% CI 0.016 to 0.072, *p* = 0.003). In the memory domain, only higher handgrip strength remained independently associated with better performance (B = 0.055, 95% CI 0.006 to 0.103, *p* = 0.028). In the language domain, both HGS (B = 0.069, 95% CI 0.012 to 0.126, *p* = 0.020) and age (B = 0.052, 95% CI 0.009 to 0.095, *p* = 0.019) were independently associated with better performance. For executive function, higher HGS was associated with better performance (B = 0.050, 95% CI 0.013–0.086, *p* = 0.009), and age was also associated with executive function scores (B = 0.053, 95% CI 0.026 to 0.081, *p* = 0.001). Finally, both baseline HGS and age were independently associated with global cognitive performance (B = 0.053, 95% CI 0.016 to 0.089, *p* = 0.006; B = 0.037, 95% CI 0.009 to 0.054, *p* = 0.010).

## 4. Discussion

This study reveals that in the context of MBS, muscle strength plays a more prominent role than muscle mass in predicting cognitive performance 12 months post-surgery. This finding aligns with recent studies suggesting that muscle strength is a more robust predictor of cognitive function than the mere measurement of muscle mass [32,33]. The fact that muscle strength was significantly associated with performance in multiple cognitive domains suggests that functional muscle capabilities, such as strength, are a better reflection of neuromuscular health and, therefore, cognitive function compared to the amount of muscle mass alone.

In our study, ALM decreased and HGS remained unchanged 12 months after MBS; however, when these parameters were adjusted for body size, both showed an improvement, in line with findings from a previous study [36]. The most recent ESPEN and EASO consensus for the definition of sarcopenic obesity supports the idea of normalizing skeletal muscle mass and HGS to body size, as these indices better reflect relative muscle adequacy and functionality in individuals with obesity. While absolute muscle mass may appear preserved or even elevated due to excess fat mass, functional impairments can still be present. These normalized parameters aim to capture such deficits more accurately, particularly in the context of MBS. The consensus also recommends the assessment of body composition using DXA as a first choice and the bioelectrical impedance analysis (BIA) as an alternative second choice [37].

Our results highlight that MBS significantly improves cognitive performance in specific domains, such as attention, memory, language, and executive function, while no significant changes were observed in the psychomotor and visuospatial domains. In addition, the global composite score, representing overall cognitive performance, also showed significant improvement. However, the effects of MBS on cognitive outcomes, especially in specific cognitive domains, have been inconsistently reported across the literature. Some studies have shown improvements in cognitive performance following MBS, particularly in attention and memory [28], while others have found no significant cognitive changes [30]. These discrepancies in the literature may be attributed to several factors, including methodological variations in study design, sample characteristics and assessment tools used. Different studies use various batteries of neurocognitive tests, which may assess cognitive domains in different ways. Some tests may be more sensitive to changes in specific domains, while others may miss subtle changes. Differences in how cognitive impairment is measured and classified can contribute to divergent results in the literature. The time frame for cognitive assessments following MBS is another important factor contributing to the inconsistency in results. Some studies assess cognitive performance immediately after surgery or within a short period of time (e.g., 3 months), while others evaluate long-term cognitive changes over a year or more. Cognitive changes following surgery may take time to manifest, and earlier studies may not capture the full extent of improvements. For instance, our study assessed cognitive function 12 months after surgery, allowing for a longer follow-up period, which may have captured more significant changes. Additionally, shorter follow-up periods may be affected by practice effects, particularly in neurocognitive tests that are repeated over time, potentially leading to artificially inflated results. However, in our study, parallel forms were used when available to further reduce practice effects, specifically for the FCSRT, forward and backward digit span, BNT and ROCF.

Another interesting finding was the improvement in QoL at 12 months post-surgery, which correlates with changes in body composition, including reductions in body fat and improvements in muscle mass and strength. Our findings reinforce the notion that MBS not only improves physical health but also has a positive impact on psychological well-being and cognitive function.

To our knowledge, no specific cut-off values for HGS have been established in MBS populations. However, a study conducted in a Chinese cohort with mild cognitive impairment (MCI) and Alzheimer’s disease (AD) proposed exploratory thresholds for HGS using ROC analysis; for individuals under 70 years, the cut-off values associated with cognitive impairment were 21.9 kg for females and 36.2 kg for males. These values may serve as a preliminary reference for identifying individuals at risk of cognitive decline and support the potential utility of HGS as a screening tool in broader clinical settings [38].

The implication of our findings is that they highlight the need to integrate routine physical function assessment into the preoperative evaluation of patients with severe obesity. The measurement of HGS offers a highly practical and cost-effective alternative. This simple and non-invasive test can be easily performed in a variety of clinical settings without the need for advanced equipment. Identifying patients at higher risk for cognitive decline could guide the development of targeted interventions, such as cognitive training and physical rehabilitation, to optimize postoperative outcomes. Strength training, as part of a comprehensive approach to treating obesity and improving metabolic outcomes, could provide a dual benefit: enhancing physical health while also promoting long-term cognitive function.

This study has several limitations that should be considered when interpreting the results. First, the sample size is relatively small, which may limit the generalizability of the findings. A larger cohort would be necessary to increase statistical power and ensure the robustness of the conclusions. Second, the observational nature of the study prevents us from establishing causal relationships between muscle strength, body composition, and cognitive outcomes. Although we controlled for potential confounders, residual confounding may still exist, particularly in variables not included in the analysis. For instance, other factors such as psychosocial variables, sleep quality and medication use, which could influence cognitive function, were not assessed. Third, the study only included a 12-month follow-up period, and it remains unclear whether the observed improvements in cognitive function are sustained in the long term. Future studies with longer follow-up durations are necessary to determine whether these cognitive changes persist. Lastly, the absence of a control group limits the ability to definitively attribute the observed cognitive improvements solely to bariatric surgery. Including a control group would allow for better comparisons and more robust conclusions.

## 5. Conclusions

In conclusion, this study highlights the significant role of muscle strength, measured via HGS, in predicting cognitive outcomes 12 months after MBS. The study also demonstrates that MBS can lead to improvements in cognitive performance, particularly in domains like attention, memory and language. These findings underscore the need for further research into the complex relationship between muscle function, body composition and cognition in bariatric surgery patients, with the aim of improving both physical and cognitive health outcomes.

## Figures and Tables

**Table 1:** Baseline characteristics of the patients included in the study.

	Total (N = 55)
Age, years	52 ± 9
Female, n (%)	41 (74.5)
Education, n (%)	
Low (≤8 years)	23 (41.8)
Medium–high (≥9 years)	32 (58.2)
Diabetes, n (%)	26 (47.3)
Dyslipidemia, n (%)	24 (43.6)
Hypertension, n (%)	33 (60)
Sleep apnea, n (%)	14 (25.5)

**Table 2:** Laboratory parameters: lipid and glycemic profile.

Total (N = 55)	Baseline	12 Months	* *p*-Value
Total cholesterol (mg/dL)	177.30 ± 34.95	153.05 ± 25.08	<0.001
HDL-cholesterol (mg/dL)	47.58 ± 11.97	56.08 ± 13.21	<0.001
LDL-cholesterol (mg/dL)	101.58 ± 31.9	79.65 ± 21.64	<0.001
Triglycerides (mg/dL)	127.35 (90.35–191.45)	78.75 (66.06–97.12)	<0.001
Plasma glucose (mmol/L)	6.09 (5.39–8.50)	4.58 (4.26–5.29)	<0.001
Insulin (pmol/L)	19.55 (13.90–31.90)	5.77 (3.94–8.00)	<0.001
HOMA-IR	5.98 (3.71–10.95)	1.16 (0.78–1.94)	<0.001
HbA1c (%)	6.15 (5.47–7.12)	5.40 (5.10–5.70)	<0.001

Values are expressed as mean ± SD, median (IQR), or n (%) * *p* < 0.05. HOMA-IR: homeostatic model assessment for insulin resistance; HbA1c: glycated hemoglobin.

**Table 3:** Body composition and quality of life.

	Baseline	12 Months	* *p*-Value
Body composition			
BMI, mean (SD)	43.53 ± 4.88	30.33 ± 3.68	<0.001
Trunk fat mass (Kg)	32.02 ± 5.25	17.16 ± 5.61	<0.001
Total fat mass (%)	53.0 (48.1–56.0)	40.7 (35.3–47.5)	<0.001
ALM	18.98 (16.70–21.63)	16.94 (14.90–20.55)	<0.001
ALM/W	17.85 ± 2.93	23.06 ± 3.64	<0.001
Dynamometer	25.4 (20.92–34.17)	25.5 (21.55–33.7)	0.735
Dynamometer/BMI	0.48 (0.41–0.52)	0.70 (0.57–0.92)	<0.001
QoL	−0.25 (−0.75–0.75)	2.50 (1.75–3.00)	<0.001

Values are expressed as mean ± standard deviation and n (%). BMI: body mass index; ALM: appendicular lean mass; ALM/W: appendicular lean mass adjusted by weight; QoL: quality of life (Moorehead–Ardelt questionnaire); * *p* < 0.05.

**Table 4:** Neuropsychological results.

Neurocognitive Test	Domain	Baseline	12 Months	*p*-Value *
Digit Span Forward	Attention/ Processing Speed	−0.25 ± 0.72	0.10 ± 0.71	<0.001
Corsi Block Forward				
TMT A				
SDMT				
ROCF-Time				
FCSRT-TFR	Memory	0.07 ± 0.88	0.67 ± 0.82	<0.001
FCSRT-TR				
FCSRT-TDFR				
FCSRT-TDR				
ROCF-3 min				
ROCF-30 min				
GPT-Right	Psychomotor Speed	−0.27 ± 1.48	−0.05 ± 1.51	0.119
GPT-Left				
BNT	Language	0.34 ± 1.22	0.58 ± 1.09	0.003
Digit Span Backward	Executive Function	−0.33 ± 0.67	−0.12 ± 0.71	<0.001
Corsi Block Backward				
TMT B				
Block Design				
Stroop Color-Word				
Verbal Fluency-Semantic				
Verbal Fluency-Phonemic				
ROCF-Copy	Visuospatial Praxis	0.18 ± 0.98	0.12 ± 0.92	0.658
	Global Composite	−0.06 ± 0.70	0.21 ± 0.67	<0.001

TMT, Trail Making Test; SDMT, Symbol Digit Modalities Test; FCSRT, Free and Cued Selective Reminding Test; TFR, Total Free Recall; TR, Total Recall; TDFR, Total Delayed Free Recall; TDR, Total Delayed Recall; ROCF, Rey–Osterrieth Complex Figure; GPT, Grooved Pegboard Test; BNT, Boston Naming Test. * *p* < 0.05.

## Data Availability

The data presented in this study are available on request from the corresponding author due to ethical and privacy restrictions, as they contain confidential clinical information.

## Abbreviations

The following abbreviations are used in this manuscript:

MBS	Metabolic Bariatric Surgery
SO	Severe Obesity
BMI	Body Mass Index
DXA	Dual-energy X-ray absorptiometry
HGS	Handgrip Strength
NTB	Neurocognitive Test Battery
QoL	Quality of Life
SDMT	Symbol Digit Modalities Test
TMT	Trail Making Test
BNT	Boston Naming Test
FCSRT	Free and Cued Selective Reminding Test
ROCF	Rey- Osterrieth Complex Figure
GPT	Grooved Pegboard Test
ALM	Appendicular Lean Mass
ALM/W	Appendicular Lean Mass adjusted by Weight
HOMA-IR	Homeostatic model assessment for insulin resistance
